# Recognition of regions of stroke injury using multi-modal frequency features of electroencephalogram

**DOI:** 10.3389/fnins.2024.1404816

**Published:** 2024-06-10

**Authors:** Yan Jin, Jing Li, Zhuyao Fan, Xian Hua, Ting Wang, Shunlan Du, Xugang Xi, Lihua Li

**Affiliations:** ^1^Institute of Intelligent Control and Robotics, Hangzhou Dianzi University, Hangzhou, China; ^2^Jinhua People’s Hospital, Jinhua, China; ^3^Affiliated Dongyang Hospital of Wenzhou Medical University, Dongyang, China

**Keywords:** stroke, regions of brain injury, EEG, wavelet packet transform, functional connectivity, feature fusion

## Abstract

**Objective:**

Nowadays, increasingly studies are attempting to analyze strokes in advance. The identification of brain damage areas is essential for stroke rehabilitation.

**Approach:**

We proposed Electroencephalogram (EEG) multi-modal frequency features to classify the regions of stroke injury. The EEG signals were obtained from stroke patients and healthy subjects, who were divided into right-sided brain injury group, left-sided brain injury group, bilateral brain injury group, and healthy controls. First, the wavelet packet transform was used to perform a time-frequency analysis of the EEG signal and extracted a set of features (denoted as WPT features). Then, to explore the nonlinear phase coupling information of the EEG signal, phase-locked values (PLV) and partial directed correlations (PDC) were extracted from the brain network, and the brain network produced a second set of features noted as functional connectivity (FC) features. Furthermore, we fused the extracted multiple features and used the resnet50 convolutional neural network to classify the fused multi-modal (WPT + FC) features.

**Results:**

The classification accuracy of our proposed methods was up to 99.75%.

**Significance:**

The proposed multi-modal frequency features can be used as a potential indicator to distinguish regions of brain injury in stroke patients, and are potentially useful for the optimization of decoding algorithms for brain-computer interfaces.

## 1 Introduction

The global death rate from stroke (stroke, cerebral infarction) has been slowly declining over the past 20 years, but the total number of patients who suffer a stroke and the total number of stroke-related deaths worldwide is huge and still increasing each year. Tsao et al. (2022) have recently shown that stroke has become the second leading cause of death worldwide.

Electroencephalogram (EEG) is a method of recording brain activity using electrophysiological indicators that allows for low-cost, continuous, real-time, non-invasive monitoring of brain function. It has been demonstrated that EEG can be used effectively to detect a variety of brain-related activities, such as staging of sleep, diagnosis of seizures, determination of the degree of coma, and cognitive and emotional monitoring (Röschke and Aldenhoff, 1992). As a large proportion of stroke patients are middle-aged or elderly, and as stroke is often characterized by rapid onset and deterioration, it is important that stroke patients are diagnosed quickly and correctly so that they can receive early and effective treatment to prevent deterioration of their condition (Savelov et al., 2019). It is important to improve the accuracy and efficiency of stroke diagnosis if the EEG signals of stroke patients can be quickly classified and the location of intracranial lesions can be effectively classified.

EEG is now increasingly becoming an important tool for the detection of stroke and the location of intracranial lesions in the brain. In recent years, with the rapid development of science and technology, EEG signals are playing an increasingly important role in the diagnosis and classification of brain diseases. Studies of the EEG characteristics of stroke patients have shown that variations occur within four frequencies: α (8–13 Hz), β (14–30 Hz), δ (1–3 Hz), and θ (4–7 Hz) bands. Schneider et al. analyzed EEG frequencies and brainwave topography in 20 patients with mild stroke (Schneider and Jordan, 2005). Their results confirmed a significant decrease in α band and an increase in δ band activity in 17 patients with mild stroke. In another study, Finnigan et al. suggested that compared to healthy individuals, stroke patients had much higher δ band and much lower α and β bands (Finnigan et al., 2016). In addition, compared to normal subjects, δ and θ bands in stroke patients show a stronger pathophysiological and slow activity profile, whereas α and β bands show faster activity. Studies using frequency extraction techniques are useful, and several studies have previously used wavelet extraction to determine significant changes in EEG signals in stroke patients (Djamal et al., 2019).

Wijaya et al. proposed a method to detect ischemic stroke disease by various signal processing and machine learning techniques (Wijaya et al., 2015). EEG data were processed by various signal processing such as fast Fourier transform (FFT), wavelet transform (WT), short time Fourier transform (SFFT), and power spectral density (PSD), followed by multi-layer perceptron (MLP) and Decision tree techniques are used to detect ischemic strokes. Djamal et al. proposed a method for identifying stroke patients using convolutional neural networks (CNN) (Djamal et al., 2020). They used wavelet decomposition to extract α, β, θ, δ, and μ bands of the raw EEG signal as features to classify stroke patients and healthy individuals. The accuracy of the test data was 90% with amplitude and β features while it was 70% without. Giri et al. conducted a stroke identification study using a one-dimensional CNN and batch normalization method based on EEG and electro-oculogram (EOG) signals (Giri et al., 2016). The collected data consisted of the relative values, correlation aspects, variance, spectral mean, entropy, kurtosis and fractal index of the EEG, and the final F-Score was 86.1%. Vivaldi et al. used spectral analysis techniques to process the raw signals, extracting spectral features including phase amplitude coupling (PAC), absolute and relative power spectral density (PSD) within the frequency band, spectral entropy (SE), and inter-channel coherence (COH) (Vivaldi et al., 2021). Patients with cranio-cerebral injury, stroke patients and healthy individuals were classified with an accuracy of 85%. A study using wavelet packet decomposition and log-energy entropy for EEG signal self-regulation by brain-computer interface EEG analysis using a multilayer perceptron to classify the 2003 BCI competition dataset yielded an accuracy of 92.83% for la and 63.33% for lb (Göksu, 2018). The above-mentioned studies extracted certain features of EEG for classification, focusing only on one type of EEG features, ignoring the correlation between different indicators of EEG, thus not making good comprehensive use of multiple aspects of EEG features.

The current research on EEG signals in stroke is focused on motor imagery, emotional classification and the diagnosis of post-stroke depression. Stroke rehabilitation systems based on EEG-BCI are built to manipulate the movement posture of rehabilitation devices (e.g., the patient’s motor imagery to realize the manipulation of a prosthesis) by decoding the patient’s motor imagery EEG signals as control commands (Fan et al., 2023; Huang et al., 2023). This type of rehabilitation system increases the patient’s involvement in the stroke rehabilitation process and can significantly shorten the rehabilitation process. Some of the EEG decoding algorithms used in these systems enable the classification of motor intentions by recognizing discriminative features between brain regions of the user during motor imagery [e.g., Common spatial pattern algorithms (Ang et al., 2008)]. However, due to the presence of brain region lesions in stroke patients and the different categories of patients with different brain region lesions, decoding algorithms traditionally applied to the general population should therefore perform poorly in stroke patients.

In this work, we propose a method to differentiate regions of brain injury by fused wavelet packet transform features and functional connectivity features for use. We first analyze the two features individually and then fuse the features to calculate their classification accuracy. It is demonstrated experimentally that the features obtained by this method are more accurate than other features for regions of brain injury differentiation. A potential significance of this study is to identify the current patients’ brain region lesions, and to use the identification results as constraints for traditional EEG decoding algorithms to guide the design of EEG decoding algorithms for stroke patients. Besides, the second potential significance of this study is to design a portable home EEG monitoring device to determine the stroke rehabilitation process of patients from the perspective of changes in the functional brain network.

## 2 Materials and methods

### 2.1 Subjects

The current work was carried out with the approval by the Ethics Committee of Dongyang People’s Hospital and in accordance with the Declaration of Helsinki. This study included 23 patients and 10 healthy subjects providing informed consent, Table 1 records demographic information for all patients. The subjects were divided into four groups as follows.

**Table 1:** Demographics of patients.

Groups (Counts)	Age	Gender	Regions of brain injury	Consciousness	Hemiplegia side
PR (8)	43	M	Right frontal lobe	Cognitive impairment	Extremities
	47	M	Right basal ganglia, right thalamus, right occipital-parietal lobe, multiple contusion and laceration of brain in corpus callosum	Unconsciousness	Extremities
	70	F	Right basal ganglia	Unconsciousness	Extremities
	55	M	Right frontal lobe, right basal ganglia, right center semioval, multiple contusion and laceration of brain in corpus callosum	Unconsciousness	Left
	28	M	Right frontal-temple lobe, right basal ganglia	Clear	Right
	44	M	Right basal ganglia extra-capsular region	Cognitive impairment	Left
	33	F	Right basal ganglia	unconsciousness	Extremities
	44	M	Right cerebellum	Cognitive impairment	Right
PL (8)	63	F	Left basal ganglia	Clear	Right
	45	M	Left frontal lobe, left basal ganglia	Clear	Right
	38	M	Left temple- occipital lobe, multiple contusion injury in left cerebellar hemisphere	Unconsciousness	Extremities
	57	F	Left temporal lobe and lateral ventricle	Cognitive impairment	Right
	44	M	Left basal ganglia	Unconsciousness	Right
	30	M	Left basal ganglia	Clear	Right
	52	F	Left frontal lobe, left basal ganglia	Clear	Right
	56	F	Left temporal–parietal occipital lobe	Cognitive impairment	Extremities
PB (7)	62	M	Bilateral frontal lobes	Cognitive impairment	Right
	54	M	Bilateral frontotemporal parietal and subdural	Unconsciousness	Extremities
	49	M	Bilateral basal ganglia	Unconsciousness	Extremities
	21	M	Bilateral hemispheres are symmetrical, bilateral gray matter, brain stem and cerebellum density are generally low.	Unconsciousness	Extremities
	25	M	Bilateral frontal lobes and pons	Clear	Left
	47	F	Bilateral temporal lobes, bilateral semioval centers	Unconsciousness	Extremities
	35	M	Bilateral basal ganglia	Unconsciousness	Extremities

Right-sided brain injury group (PR group): patients with the regions of brain injury located on the right side of the brain.

Left-sided brain injury group (PL group): patients with the regions of brain injury located on the left side of the brain.

Bilateral brain injury group (PB group): patients with the regions of brain injury in both the left and right side of the brain.

Healthy control group (HC group): Ten healthy subjects, all right-handed (age: 24–27 years; 3 females, 7 males). None of these healthy subjects had neurological or musculoskeletal disorders and were not taking prohibited drugs.

### 2.2 EEG recordings and preprocessing

**Figure 1:**
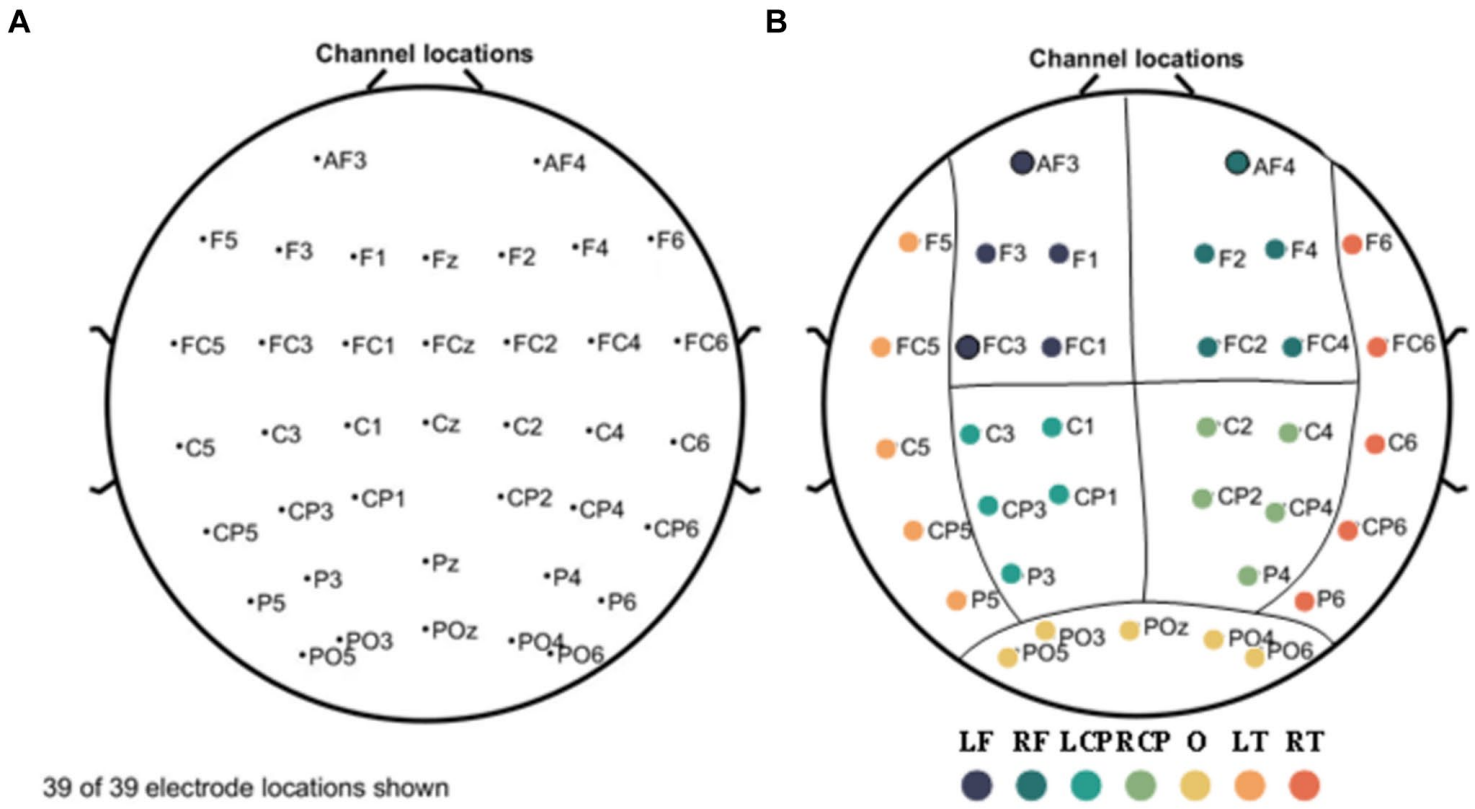
Electrode names and positions on the head **(A)** and seven scalp regions **(B)**. The measurement region is divided into seven regions: left frontal (LF); right frontal (RF); left central-parietal (LCP); right central-parietal (RCP); occipital (O); left temporal (LT); and right temporal (RT) regions, represented by separate colors, respectively.

EEG data were collected continuously for 3 min with the subjects in a relaxed closed-eye (1.5 min) and open-eye state (1.5 min). A 64-channel wireless EEG system (NeuSen.W64, Neuracle, China) was used to collect EEG data with a sampling frequency of 1,000 Hz. Referring to the international 10–20 system, as shown in Figure 1A, we selected 39 channels from the 64 EEG channels for measurement (the reference electrode is located in the middle of Cp1 and Cp2, the ground electrode is located in the middle of AF3 and AF4, and GREENTECH’s GT5 medical conductive gal is used). We eliminated the four electrodes located in the median line: Fz, FCz, Cz, and Pz, according to the arrangement and position of the 39 channels on the scalp, and grouped the remaining channels into seven scalp regions to obtain a regional analysis (Figure 1B). These regions include: left frontal (LF); right frontal (RF); left central-parietal (LCP); right central-parietal (RCP); occipital (O); left temporal (LT) and right temporal (RT). Features were extracted from each of the channels used for analysis. Each feature was averaged for each region and used to analyze differences between groups.

The raw EEG signal was downsampled to 250 Hz using the EEGLAB toolbox and further performed bandpass filtering from 0 to 32 Hz. In addition, Cleanline (an EEGLAB plug-in) is used to remove sinusoidal artifacts in the scalp channel that are not effectively removed by trap filtering. The combination of AC power line fluctuations, equipment power and fluorescent lamps may produce sinusoidal artifacts. Other artifacts, such as ECG, EMG and EOG, are removed by technical independent component analysis (ICA) using the Infomax algorithm in EEGLAB (the probability of an artifact is first given by the program, and then further eliminated by an experienced expert). The artifact-free signal is then denoised using the wavelet transform. This is an acceptable combination of ICA and wavelet denoising for removing noise from EEG signals (Mahajan and Morshed, 2014). For the activity segment (1.5 min) captured by each subject, this paper uses 30s as the window width and 2 s as the interval, and utilizes the sliding window method to segment the activity segment into 31 subsamples, which ultimately constitute the sample set for that subject. The EEG data were processed and analyzed in the MATLAB (version 8.2.0.701.R2017a).

## 3 Analysis methods

### 3.1 Time-frequency analysis

#### 3.1.1 Wavelet packet transform

Wavelet packet transform is a modern time-frequency analysis and processing method that can effectively process all kinds of non-smooth random signals. In this paper, db3 is used as the wavelet basis function for wavelet packet decomposition, and the signal is decomposed in the frequency domain by five layers of wavelet packets to obtain a spectral analysis of the signal. Given the Eth epoch in the analysis, the corresponding time-frequency representation is estimated for each EEG channel.

#### 3.1.2 Wavelet packet transform-based features (WPT features)

The spectrum-based features are explained below:

- Energy Ratio

The wavelet packet decomposition was applied to decompose the collected EEG signal. Get the δ [1-3 Hz], θ [4–7 Hz], α_1_ [8–10 Hz], α_2_ [10–13 Hz], β_1_ [14–20 Hz] and β_2_ [21–30 Hz] frequency bands and calculate the energy carried by the signal in that band range. The energy of the signal at different decomposition scales is defined as Equation (1):


(1) $$E_{j,i}=\underset{k\in Z}{\sum }{\left[p_{s}\left(n,j,k\right)\right]}^{2}$$

where $E\left(j,i\right)$ denotes the energy value of the ith node at decomposition level *j*; $p_{s}\left(n,j,k\right)$ is the wavelet packet transform coefficient. To facilitate the calculation, we have considered normalizing the energy of each node, i.e., taking the percentage of the energy of each node as Equation (2):


(2) $$ER=\frac{E_{j,i}}{E}\times 100\%$$

where *E* represents the sum of the energy obtained from the six bands decomposed.

The energy ratio obtained for each channel in the same frequency band is expressed in the form of a matrix as Equations (3–5):


(3) $$\begin{array}{l} \delta =\left[\begin{array}{cccc} ER\delta_{1} & 0 & \cdots & 0\\ 0 & ER\delta_{2} & \cdots & 0\\ \cdots & \cdots & \cdots & \cdots \\ 0 & 0 & \cdots & ER\delta_{n} \end{array}\right]\;\\ \theta =\left[\begin{array}{cccc} ER\theta_{1} & 0 & \cdots & 0\\ 0 & ER\theta_{2} & \cdots & 0\\ \cdots & \cdots & \cdots & \cdots \\ 0 & 0 & \cdots & ER\theta_{n} \end{array}\right] \end{array}$$

(4) $$\begin{array}{l} \alpha_{1}=\left[\begin{array}{cccc} ER\alpha_{11} & 0 & \cdots & 0\\ 0 & ER\alpha_{12} & \cdots & 0\\ \cdots & \cdots & \cdots & \cdots \\ 0 & 0 & \cdots & ER\alpha_{1n} \end{array}\right]\;\\ \alpha_{2}=\left[\begin{array}{cccc} ER\alpha_{21} & 0 & \cdots & 0\\ 0 & ER\alpha_{22} & \cdots & 0\\ \cdots & \cdots & \cdots & \cdots \\ 0 & 0 & \cdots & ER\alpha_{2n} \end{array}\right] \end{array}$$

(5) $$\begin{array}{l} \beta_{1}=\left[\begin{array}{cccc} ER\beta_{11} & 0 & \cdots & 0\\ 0 & ER\beta_{12} & \cdots & 0\\ \cdots & \cdots & \cdots & \cdots \\ 0 & 0 & \cdots & ER\beta_{1n} \end{array}\right]\;\\ \beta_{2}=\left[\begin{array}{cccc} ER\beta_{21} & 0 & \cdots & 0\\ 0 & ER\beta_{22} & \cdots & 0\\ \cdots & \cdots & \cdots & \cdots \\ 0 & 0 & \cdots & ER\beta_{2n} \end{array}\right] \end{array}$$

Where *ERδ_j_*, *ERθ_j_*, *ERα_1j_*, *ERα_2j_*, *ERβ_1j_*, *ERβ_2j_* denote the energy ratio of the *j*th channel in the δ, θ, α_1_, α_2_, β_1_, β_2_ bands respectively, and the matrices are all 39 × 39 two-dimensional square arrays.

- Wavelet Entropy

Wavelet entropy is extracted to characterize the irregularity of brain by assessing the disorder and diversity of the EEG as shown in Equations (6–11).

- Wavelet energy entropy (WEE)

Wavelet energy entropy quantifies the energy distribution of a complex time-varying signal in each time-frequency space from a macroscopic perspective. The relative energy of each band is:


(6) $$e_{j,i}=\frac{E_{j,i}}{\sum_{j=0}^{2^{i-1}}E_{j,i}}$$

The wavelet energy entropy is then defined as:


(7) $$\mathrm{WE}=-\overset{2^{j}-1}{\underset{j=0}{\sum }}e_{j,i}\ln\left(e_{j,i}\right)$$

Normalization of wavelet energy entropy:


(8) $$\mathrm{WEE}=\frac{\mathrm{WE}}{\ln6}$$

The resulting wavelet energy entropy is expressed in matrix as:


(9) $$\mathrm{WEE}=\left[\begin{array}{cccc} \mathrm{WE}E_{1} & 0 & \cdots & 0\\ 0 & \mathrm{WE}E_{2} & \cdots & 0\\ \cdots & \cdots & \cdots & \cdots \\ 0 & 0 & \cdots & \mathrm{WE}E_{n} \end{array}\right]$$

where $\mathrm{WE}E_{j}$ denotes the wavelet energy entropy obtained from the jth channel, and the matrix is a 39 × 39 two-dimensional square matrix.

- Wavelet singular entropy (WSE)

Wavelet singular entropy is an analysis method that combines wavelet multi-resolution analysis, singular value decomposition theory and information entropy principle.

The wavelet singular spectrum entropy is defined as:


(10) $$WSE=-\overset{6}{\underset{m=1}{\sum }}q_{m}\ln\left(q_{m}\right)$$

the $q_{m}$ is the percentage of singular values in the *m*th frequency band after wavelet packet reconstruction.

Express the resulting wavelet singular entropy in matrix as:


(11) $$WSE=\left[\begin{array}{cccc} WSE_{1} & 0 & \cdots & 0\\ 0 & WSE_{2} & \cdots & 0\\ \cdots & \cdots & \cdots & \cdots \\ 0 & 0 & \cdots & WSE_{n} \end{array}\right]$$

where $WSE_{j}$ denotes the wavelet singular entropy obtained from the *j*th channel, and the matrix is a two-dimensional square matrix of 39 × 39.

### 3.2 Brain network analysis

#### 3.2.1 Functional connectivity

The functional network is a measure of the inter-channel relationship in the form of a square matrix, with each element of the matrix taking values for its columns and rows corresponding to indicators of the inter-channel functional network.

#### 3.2.2 Functional connectivity features (FC features)

- Phase locking value (PLV)

The phase-locked value is a statistical data that can be used to investigate task-induced changes in the remote synchronization of neural activity performed from EEG data. The PLV describes the synchronization of the phases of the two channels in a certain frequency range. The PLV is calculated by Equation (12):


(12) $$\mathrm{pl}v_{ij}=\left|\frac{1}{N}\overset{N}{\underset{k=1}{\sum }}e^{j\left(\phi_{i}\left(t_{k}\right)-\phi_{j}\left(t_{k}\right)\right)}\right|$$

Each element in the resulting PLV matrix has a value between 0 and 1, and the PLV value between channel *i* and channel *j* is equal to the PLV value between channel *j* and channel *i*. This property is expressed in the PLV matrix as a symmetric matrix; moreover, the relationship between channel *i* and channel *i* is that between itself and before itself, in which case the two channels are considered to be completely coherent with each other, and the phases perfectly synchronized, and this property is expressed in the PLV matrix as the values on the diagonal are all 1. Therefore, it can be concluded that the matrix of PLV takes the form of Equation (13):


(13) $$PLV=\left[\begin{array}{cccc} 1 & \mathrm{pl}v_{12} & \cdots & \mathrm{pl}v_{1n}\\ \mathrm{pl}v_{12} & 1 & \cdots & \mathrm{pl}v_{2n}\\ \cdots & \cdots & \cdots & \cdots \\ \mathrm{pl}v_{1n} & \mathrm{pl}v_{2n} & \cdots & 1 \end{array}\right]$$

where the matrix is a 39 × 39 two-dimensional square matrix.

- Partial directed coherence (PDC)

PDC is a multivariate effective connectivity measure based on Granger causality, which measures the causal influence between channel signals and is therefore directional. The EEG signal is modeled using a multivariate autoregressive model (MVAM) and the order of the model is determined using the AIC criterion, then the directed information flow from channel j to channel i at frequency f, i.e., the PDC value, can be solved for by Equation (14):


(14) $$PDC\left(i,j,f\right)=\frac{\left|A_{ij}\left(f\right)\right|}{\sqrt{\sum_{k}A_{kj}^{*}\left(f\right)A_{kj}\left(f\right)}}$$

The value of $PDC\left(i,j,f\right)$ is normalized between [0 1] and indicates the proportion of signals flowing from $x_{j}$ to $x_{i}$ to all signals flowing from $x_{j}$ (Sameshima and Baccalá, 1999; Pereda et al., 2005). Larger values indicate stronger information flow from channel *j* to *i*. Then the PDC matrix is represented as Equation (15):


(15) $$PDC=\left[\begin{array}{cccc} pdc_{11} & pdc_{12} & \cdots & pdc_{1n}\\ pdc_{21} & pdc_{22} & \cdots & pdc_{2n}\\ \cdots & \cdots & \cdots & \cdots \\ pdc_{n1} & pdc_{n2} & \cdots & pdc_{nn} \end{array}\right]$$

where $pdc_{ij}$ refers to the value of PDC between the ith channel and the jth channel, and the matrix is a two-dimensional square matrix of 39 × 39.

In this paper, a proportional threshold of 15% is used in the calculation of FC features.

### 3.3 Multi-modal (WPT + FC) feature

From the introduction of subsection A and B, 14 sets of WPT features and 2 sets of FC features can be obtained. The resulting features are fused to obtain a total of 28 sets of multi-modal (WPT + FC) features:

14(# WPT Features) × 2(# FC features) = 28 features.

For the closed-eye data of a certain frequency band, 1 set of ER matrices and 2 sets of FC feature matrices can be obtained, and the ER matrices and the two FC feature matrices are subjected to matrix multiplication operations to obtain as shown in Equations (16, 17):


(16) $$\begin{array}{l} \mathrm{\delta PLV}=\\ \delta \times PLV=\left[\begin{array}{cccc} ER\delta_{1} & ER\delta_{1}\mathrm{pl}v_{12} & \cdots & ER\delta_{1}\mathrm{pl}v_{1n}\\ ER\delta_{2}\mathrm{pl}v_{12} & ER\delta_{2} & \cdots & ER\delta_{2}\mathrm{pl}v_{2n}\\ \cdots & \cdots & \cdots & \cdots \\ ER\delta_{n}\mathrm{pl}v_{1n} & ER\delta_{n}\mathrm{pl}v_{2n} & \cdots & ER\delta_{n} \end{array}\right] \end{array}$$

(17) $$\begin{array}{l} \mathrm{\delta PDC}=\\ \delta \times PDC=\left[\begin{array}{cccc} ER\delta_{1}pdc_{11} & ER\delta_{1}pdc_{12} & \cdots & ER\delta_{1}pdc_{1n}\\ ER\delta_{2}pdc_{21} & ER\delta_{2}pdc_{22} & \cdots & ER\delta_{2}pdc_{2n}\\ \cdots & \cdots & \cdots & \cdots \\ ER\delta_{n}pdc_{n1} & ER\delta_{n}pdc_{n2} & \cdots & ER\delta_{n}pdc_{nn} \end{array}\right] \end{array}$$

Similarly, multiplying the wavelet packet energy matrix of δ, θ, α_1_, α_2_, β_1_, and β_2_ frequency band with PLV and PDC matrix respectively, we can get $\mathrm{\theta PLV},\mathrm{\theta PDC},\alpha_{1}PLV,\alpha_{1}PDC,\alpha_{2}PLV,\alpha_{2}PDC,\beta_{1}PLV,\beta_{1}PDC,\beta_{2}PLV,\beta_{2}PDC\text{.}$ The resulting matrices are all 39 × 39 two-dimensional square matrices.

The WEE matrix and the WSE matrix for each frequency band are matrix multiplied with the FC feature matrix as Equations (18, 19), respectively, we get:


(18) $$\begin{array}{l} \mathrm{EEPLV}=\\ \mathrm{WEE}\times PLV=\left[\begin{array}{cccc} \mathrm{WE}E_{1} & \mathrm{WE}E_{1}\mathrm{pl}v_{12} & \cdots & \mathrm{WE}E_{1}\mathrm{pl}v_{1n}\\ \mathrm{WE}E_{2}\mathrm{pl}v_{12} & \mathrm{WE}E_{2} & \cdots & \mathrm{WE}E_{2}\mathrm{pl}v_{2n}\\ \cdots & \cdots & \cdots & \cdots \\ \mathrm{WE}E_{n}\mathrm{pl}v_{1n} & \mathrm{WE}E_{n}\mathrm{pl}v_{2n} & \cdots & \mathrm{WE}E_{n} \end{array}\right] \end{array}$$

(19) $$\begin{array}{l} \mathrm{SEPLV}=\\ WSE\times PLV=\left[\begin{array}{cccc} WSE_{1} & WSE_{1}\mathrm{pl}v_{12} & \cdots & WSE_{1}\mathrm{pl}v_{1n}\\ WSE_{2}\mathrm{pl}v_{12} & WSE_{2} & \cdots & WSE_{2}\mathrm{pl}v_{2n}\\ \cdots & \cdots & \cdots & \cdots \\ WSE_{n}\mathrm{pl}v_{1n} & WSE_{n}\mathrm{pl}v_{2n} & \cdots & WSE_{n} \end{array}\right] \end{array}$$

Similarly, the WEE and the WSE are multiplied with the PDC to obtain the EEPDC,SEPDC . The resulting matrix is a 39 × 39 two-dimensional square matrix.

### 3.4 Data set partitioning and classifier design

Resnet50 deep network can reprogram the network layers according to the residual learning function and can overcome the generalization error, overfitting, vanishing and explosion problems (ElGhany et al., 2021). Therefore, in this paper instead, the resnet50 deep network is used to construct the classifier. The input of the classifier is the features extracted in subsections A, B, C; the output is 0, 1, 2, and 3, where 0 means the subject belongs to the PR group, 1 means the subject belongs to the PL group, 2 means the subject belongs to the PB group, and 3 means the subject belongs to the HC group.

In the classifier, each convolutional layer and fully connected layer is followed by a linear unit with leakage correction (Leaky ReLU) as the activation function. The loss function of the model is chosen as the cross-entropy loss function as shown in Equation (20):


(20) $$L=-\frac{1}{N}\overset{N}{\underset{1}{\sum }}\overset{4}{\underset{i=1}{\sum }}y_{i}\log\left(p_{i}\right)$$

where $y_{i}$ refers to the real disease condition of subject *i* (the values of $y_{i}$ 0, 1, 2, and 3 refer to Group PR, Group PL, Group PB, and Group HC, respectively), and $p_{i}$ refers to the probability of subject *i* being predicted as category *i* by the neural network.

In this paper, we evaluate the classifier using 10-fold cross-validation. Since there are 31 samples per subject, there are a total of 1,023 samples. Based on these samples, the classification network was trained-evaluated using 10-fold cross validation using 102 samples for testing and 921 samples for training.

The model is trained using the Pytorch (version 1.11.0) framework based on CPU, and the optimizer is chosen as the Adam optimizer with an initial learning rate of 0.00001, and CosineAnnealing is used to adjust the learning rate. A total of 600 epochs are trained by reading all samples at a time.

A four-class classifier was used to classify the PR group, the PL group, the PB group, and the HC group, and a confusion matrix was determined and accuracy was calculated as Equation (21):


(21) $$\mathrm{Accuracy}=\frac{TP+TN}{TP+TN+FP+FN}$$

Where TP, TN, FP, FN represent the true positive, true negative, false positive, and false negative, respectively (Powers, 2020).

### 3.5 Statistical analysis

The statistical analysis software we chose was IBM SPSS statistics 23 (Datanine Software, China). One-way analysis of variance (ANOVA) tests was used to assess significant changes in the time-frequency underlying characteristics of the four groups. *p* < 0.05 was considered a statistically significant level. Bonferroni correction was applied to avoid spurious rejections when the variance was chi-squared (Cabin and Mitchell, 2000); Tamhane’s T2 method was used for comparison between groups when the variance was not chi-squared.

**Figure 2:**
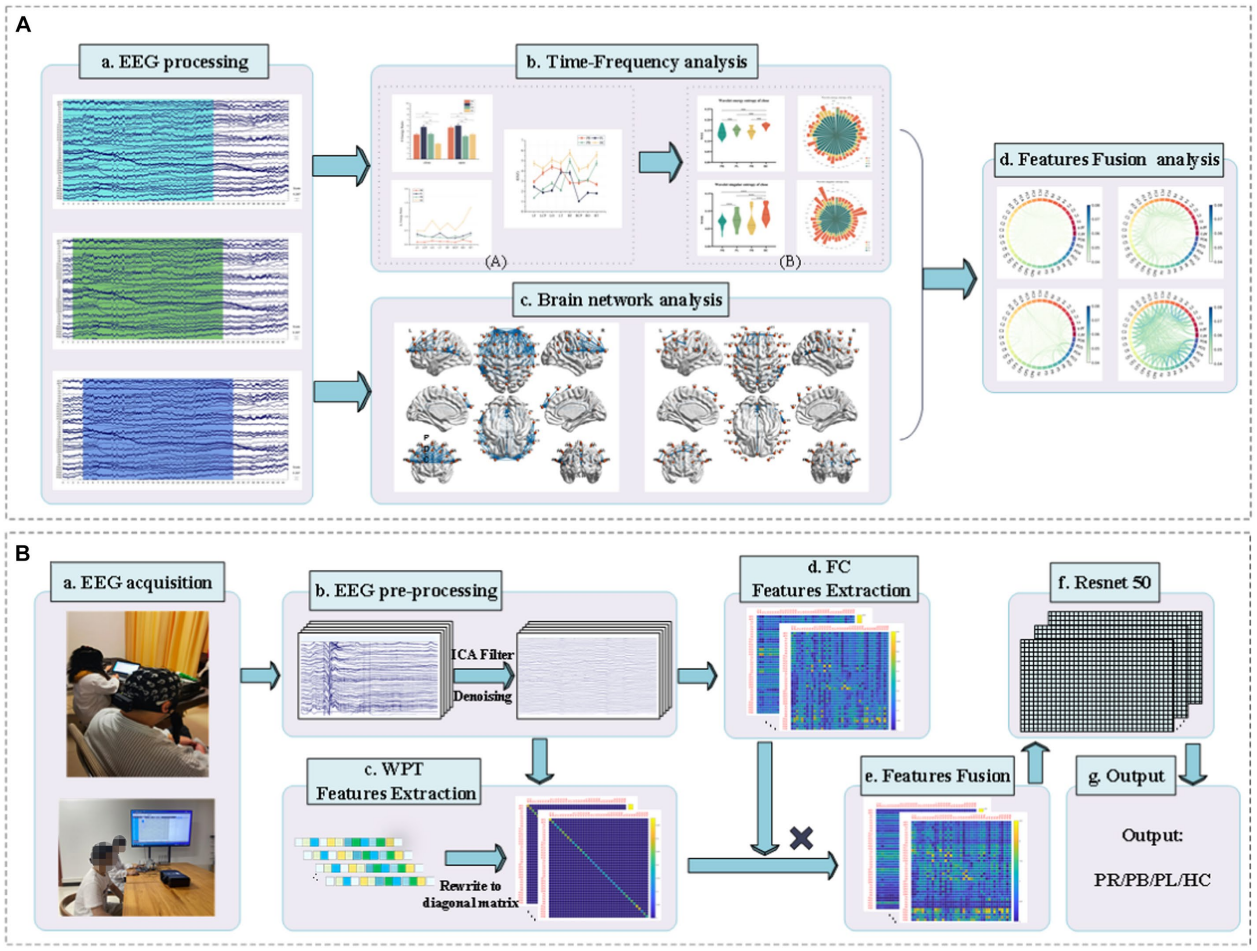
Block diagram of classification between stroke patients and the normal from the EEG analysis and classification. **(A)** EEG time-frequency analysis and brain network analysis of stroke patients and normal subjects in resting state. **(B)** Classification of regions of brain injury: (a) acquisition of EEG signals from stroke patients and normal subjects; (b) pre-processing of the acquired EEG signals; (c) extraction of WPT features, writing the features in matrix form; (d) extraction of FC features; (e) matrix product of WPT features and FC features to obtain multi-modal (WPT + FC) features; (f) feeding the multi-modal (WPT + FC) features are fed into the Resnet50 convolutional neural network for classification; (g) the final output of the classification is obtained.

Classification accuracy was used to assess the ability of functional connectivity features and multi-modal (WPT + FC) features to distinguish regions of brain injury in stroke patients. Additionally, Figure 2 shows a block diagram with the different steps followed in this study.

## 4 Results

### 4.1 Time-frequency analysis

**Figure 3:**
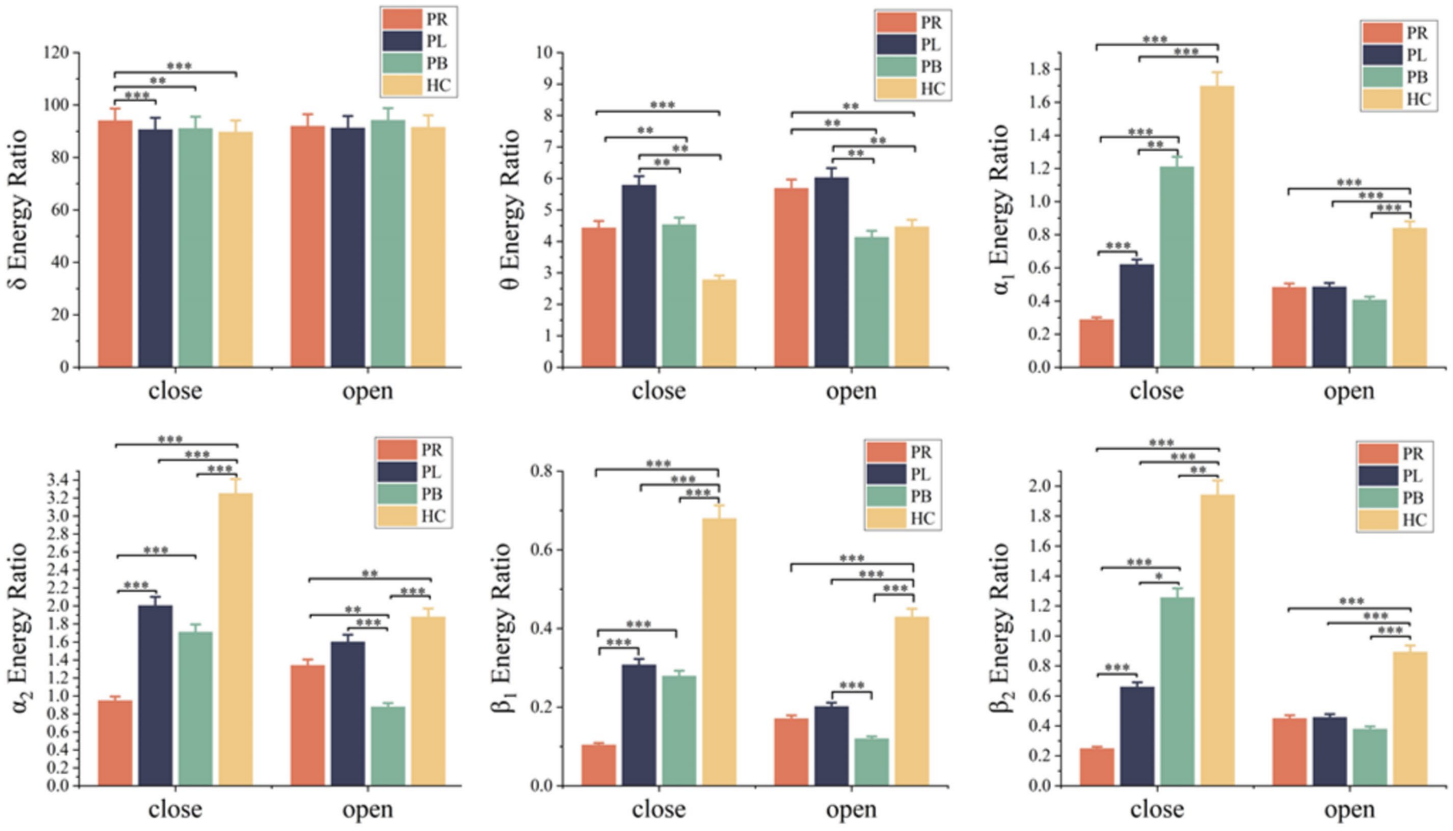
Mean δ energy ratio, mean θ energy ratio, mean α_1_ energy ratio, mean α_2_ energy ratio, mean β_1_ energy ratio and mean β_2_ energy ratio of 39 EEG channels in the open-eye (open) and closed-eye (close) states for the PR group, PL group, PB group and HC group. * denotes *p* < 0.05, ** denotes *p* < 0.01, *** denotes *p* < 0.001.

**Figure 4:**
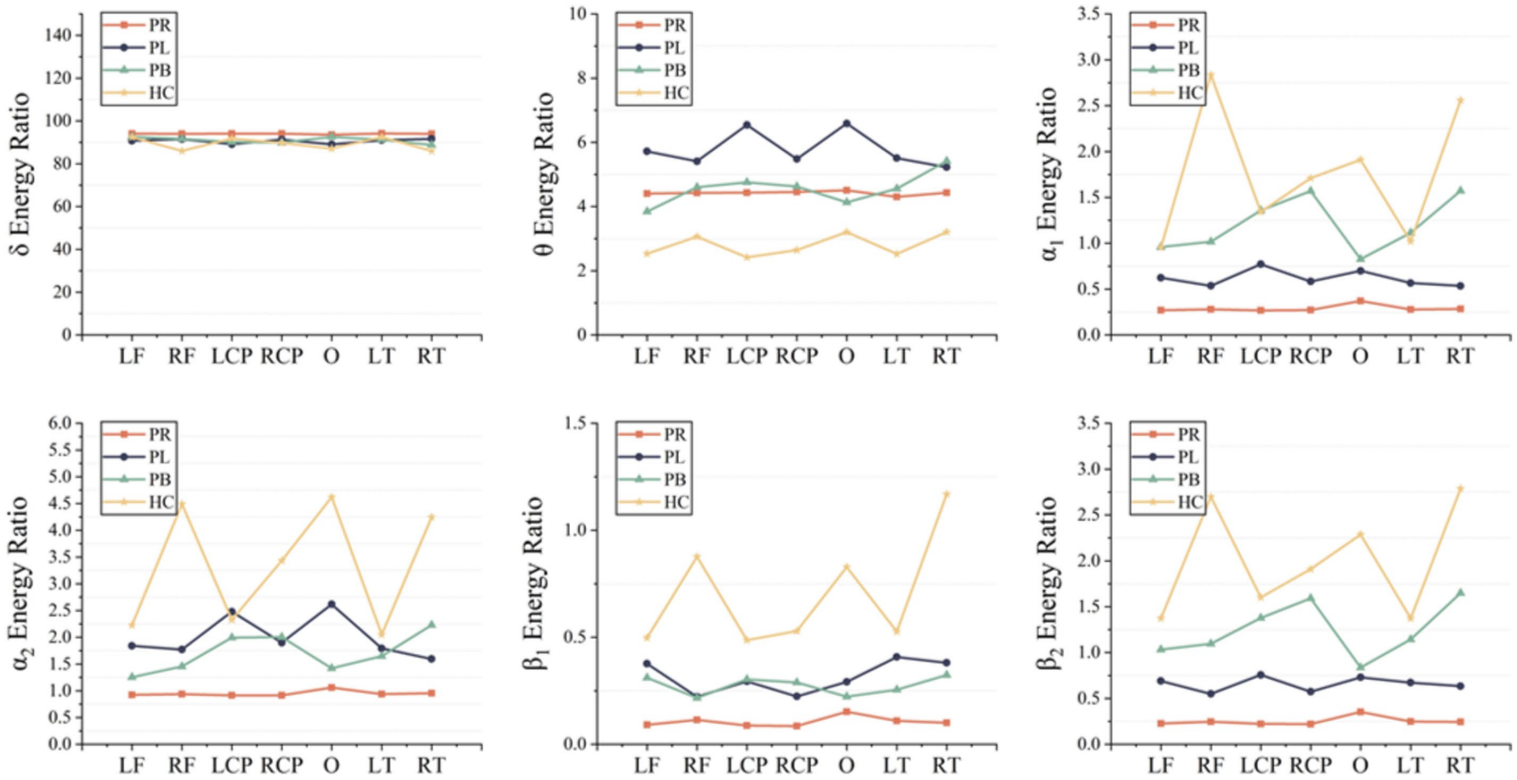
Mean δ energy ratio, mean θ energy ratio, mean α_1_ energy ratio, mean α_2_ energy ratio, mean β_1_ energy ratio and mean β_2_ energy ratio of the seven brain regions in the PR, PL, PB and HC groups in the closed (closed-eye) state.

A time-frequency analysis of the whole brain and each brain region was first performed. Figure 3 shows the average energy ratio of δ, θ, α_1_, α_2_, β_1_, and β_2_ frequency bands for 39 EEG channels in the PR group, PL group, PB group, and HC group. The energy ratios of all four groups tends to decrease with increasing frequency under both open-eye and closed-eye. The energy ratio values of δ band fluctuated between [90,95], while those of β_2_ band fluctuated between [0.25,2]. It indicates that the subjects concentrated most of their energy in the lower frequency band in the quiet state. When comparing between groups, we found that the difference between groups for each frequency band was smaller in the open-eye state than in the closed-eye state, especially between stroke patients (*p* > 0.05). Meanwhile, we can conclude from Figure 3 that in the closed-eye state, there are significant differences (*p* < 0.01) between the four groups of PR, PL, PB and HC in the α_1_, α_2_, β_1_ and β_2_ frequency bands; while in the δ and θ frequency bands, the differences between the groups are smaller. In the δ and θ frequency bands, the energy ratio of the HC group was smaller than that of the other three groups in both the open-eye and closed-eye states, while in the α_1_, α_2_, β_1_, β_2_ frequency bands, the energy ratio of the HC group was larger than that of the other three groups. Reflecting the typical slowness of brain recordings in stroke patients due to high energy in the lower frequency bands and low energy in the higher frequency bands due to brain injury. In addition, the PR group had the lowest energy ratio values in the α_1_, α_2_, β_1_, β_2_ frequency bands in the closed-eye state. Figure 4 shows the mean energy occupancy of the seven brain regions in the δ, θ, α_1_, α_2_, β_1_, and β_2_ frequency bands in the PR group, the PL group, the PB group, and the HC group with closed-eye. In the δ band, there were no significant group differences between the four groups (*p* > 0.05); in the θ band, there were no significant group differences between the PB and PL groups (p > 0.05). This is the same conclusion as that obtained in Figure 3. In the θ frequency band, stroke patients had a much larger average energy ratio across brain regions than healthy controls. In the higher frequency bands, the mean energy ratio of stroke patients was much smaller than that of HC group: the mean energy ratio of the right frontal, occipital, and right temporal in the α_1_, α_2_, β_1_, and β_2_ bands was much smaller. In the α_1_, α_2_, β_1_, and β_2_ frequency bands, the PR group had the smallest average energy ratio in each brain region. Significant differences between all four groups in RF, RCP, RT, and O brain regions in the α_1_ band (*p* < 0.01); there were significant differences between the four groups in RT and O brain regions in the α_2_ band (*p* < 0.01); Significant differences (*p* < 0.01) were found between all four groups in seven brain regions in the β_1_ and β_2_ frequency bands.

**Figure 5:**
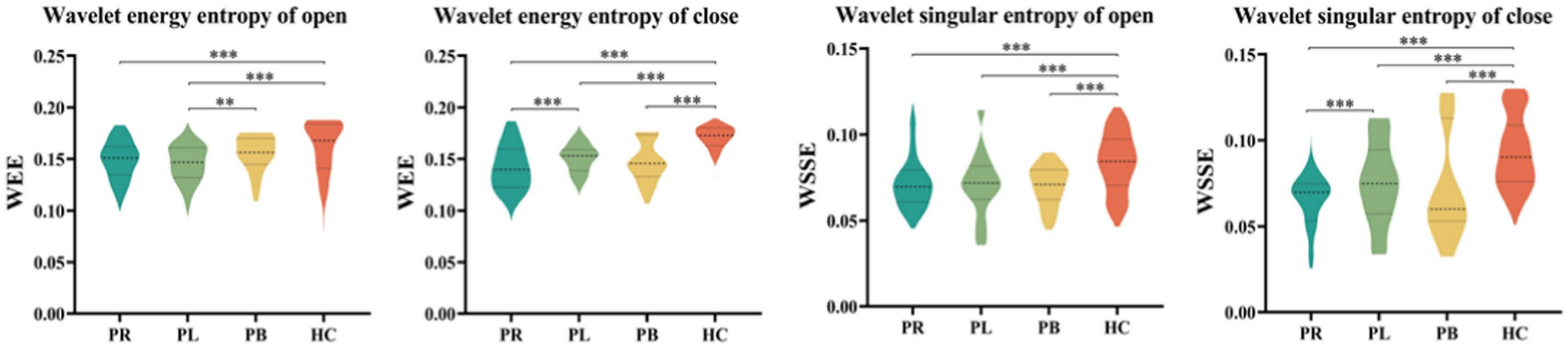
The whole-brain average wavelet energy entropy and the whole-brain average wavelet singular entropy of 39 EEG channels in the open-eye (open) and closed-eye (close) states for the PR group, PL group, PB group, and HC group.

**Figure 6:**
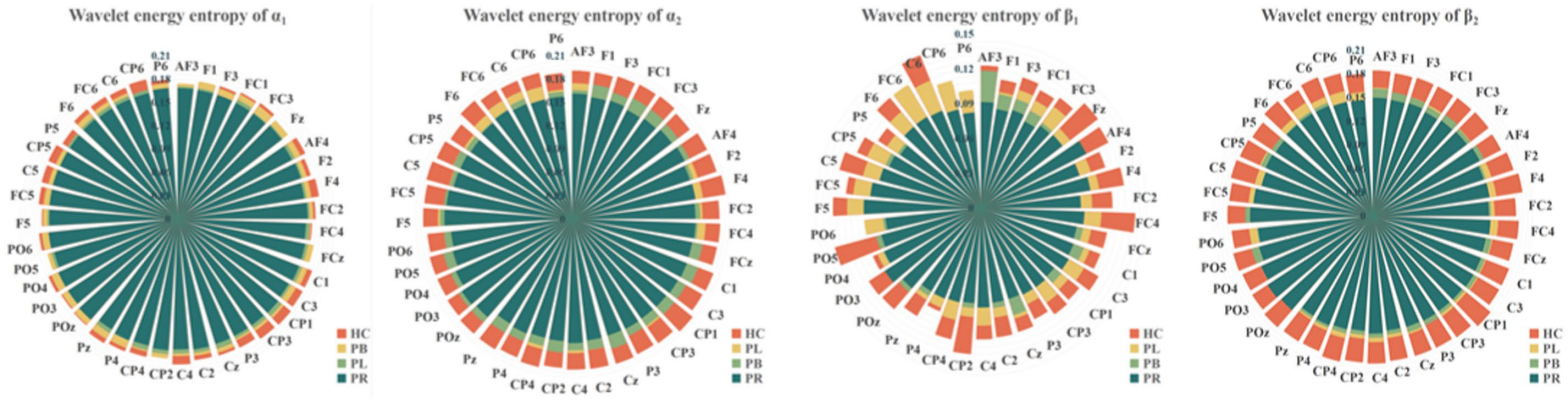
Wavelet energy entropy values for the PR, PL, PB, HC groups in the α_1_, α_2_, β_1_, and β_2_ frequency bands with closed-eye.

The whole-brain mean WEE and whole-brain mean WSE for the 39 EEG channels in the PR, PL, PB, HC groups in the open-eye and closed-eye states are given in Figure 5. From the figure we can obtain that WEE and WSE are significantly different between stroke patients and healthy controls in the two resting states, but are less able to distinguish between regions of brain injury in stroke patients. We combine the wavelet packet energy analysis for the six frequency bands mentioned above, and considering that the apparent changes occurred in the α_1_, α_2_, β_1_, and β_2_ frequency bands in the closed-eye state, we calculated the WEE values for 39 EEG channels in the α_1_, α_2_, β_1_, and β_2_ frequency bands for the four groups and applied it to the whole brain analysis, as shown in Figure 6. As we know, the wavelet energy entropy depends mainly on information about the complexity of the nonlinear dynamical process of the EEG signal and the energy distribution of each subspace signal. It was found that the WEE values of the healthy control group were greater than the other three groups in all four frequency bands, while the WEE value of the PR group was the smallest, indicating that the EEG signals acquired in the HC group were more complex than the other three groups in the closed-eye state, and the EEG signals acquired in the PR group were more single and ordered than the other three groups (Figure 6). This indicates that the irregularity of the EEG signal is significantly reduced in stroke patients. We found that WEE values in all four frequency bands were significantly different between stroke patients (PR, PL and PB groups) and HC group (*p* < 0.001). Notably, in the α_2_ band, the WEE of the FC3 channel was significantly different between the four groups (*p* < 0.01); in the β_1_ band, the WEE of the F6 and FC4 channels was significantly different between the four groups (*p* < 0.01).

**Figure 7:**
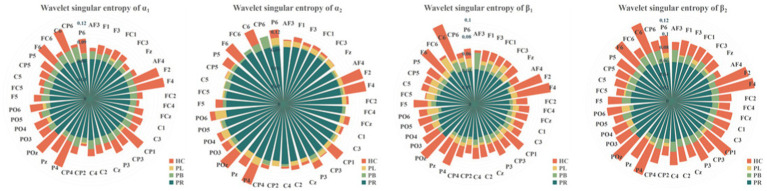
Wavelet singular entropy values for the PR, PL, PB, HC groups in the α_1_, α_2_, β_1_, and β_2_ frequency bands with closed-eye.

To show the differences between groups more clearly, we further calculated the wavelet singular entropy values for the four frequency bands in the PR, PL, PB, and HC groups in the closed-eye state, as shown in Figure 7. Similarly, the wavelet singular entropy values are larger for the HC group and smallest for the PR group in the four frequency bands. Statistical analysis allowed us to conclude that WSE values in all four frequency bands in the closed-eye state were significantly different between stroke patients (PR, PL, PB groups) and HC group (*p* < 0.001), and between the PR and PL groups (*p* < 0.01); while for the PB and PR groups, and between the PB and PL groups, there were no significant differences (*p* > 0.05). Meanwhile, we found that in the α_1_ band, the WSE of the C6 channel had significant group differences among the four groups (*p* < 0.01); in the α_2_ band, the WSE of the F1, FC3, CP1 channels had significant group differences among the four groups (*p* < 0.01); in the β_1_ band, the WSE of the FC3 and CP5 channels had significant differences among the four groups (*p* < 0.01). However, it is not clear which is more effective in classifying regions of brain injury, wavelet energy entropy or wavelet singular entropy based on frequency features. This will be followed by a categorical analysis to compare the two types of wavelet entropy.

### 4.2 Brain network analysis

**Figure 8:**
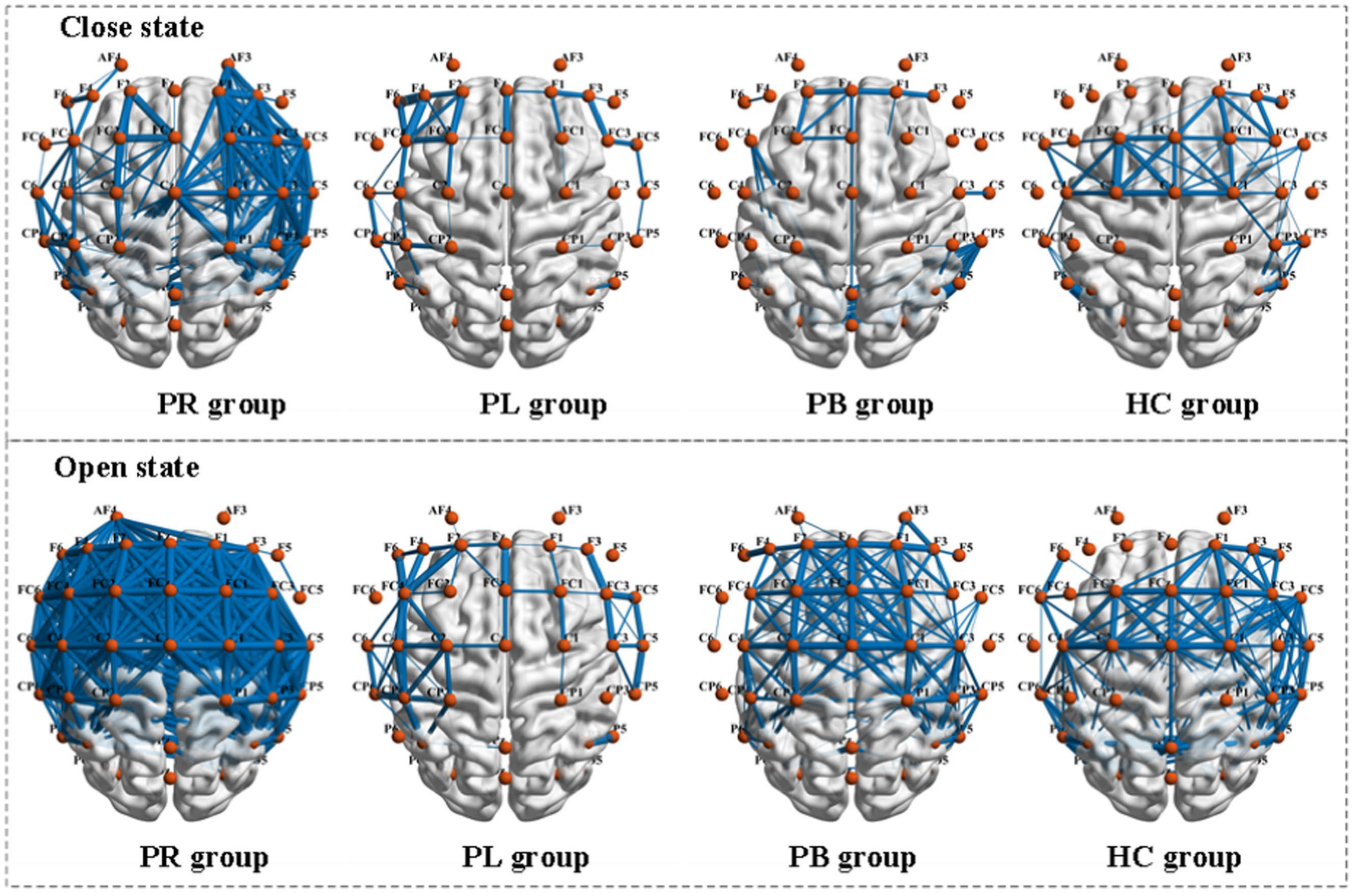
Comparison of PLV metrics between group mean channels in the PR, PL, PB, and HC groups in the closed-eye and open-eye states. The thickness of the blue line represents the size of the brain functional network values, the thicker the larger.

Functional connectivity is a measure of the relationship between channels, formally a square matrix with each element of the matrix taking values for the rows and columns corresponding to the metrics between channels. To further explore the phase coupling information of the EEG signal, we investigated the PLV values between the group average channels of the four groups in both closed-eye and open-eye state, which are presented in Figure 8. The closer the PLV value is to 1, the more synchronous the two channels are, i.e., there is a connection between the two channels. As can be seen in Figure 8, there are differences between all four groups in both the closed-eye and open-eye states. The difference between the four groups is more significant in the closed-eye state. And the differences between the PR group and the other three groups were highly significant regardless of the open-eye or closed-eye state. We further found that the synchronization between channels was stronger in the closed-eye state than in the open-eye state in the four groups. For the PR group, the synchronization between channels in the right side of the brain was stronger in the closed-eye state, whereas the synchronization between channels in the whole brain was stronger in the open-eye state. For the PL group, synchrony was stronger between channels in the left and right temporal regions in both the closed-eye and closed-eye states. For the PB group, only the synchronization between the CP5 channel and the other channels was stronger in the closed-eye state; in the open-eye state, the synchrony was stronger between channels in both frontal and central parietal regions. For the HC group, the synchronization between channels in the central parietal region and the right temporal region was stronger in the closed-eye and open-eye states. Therefore, calculating PLV values may be a sensitive analysis method to distinguish regions of brain injury in stroke patients.

**Figure 9:**
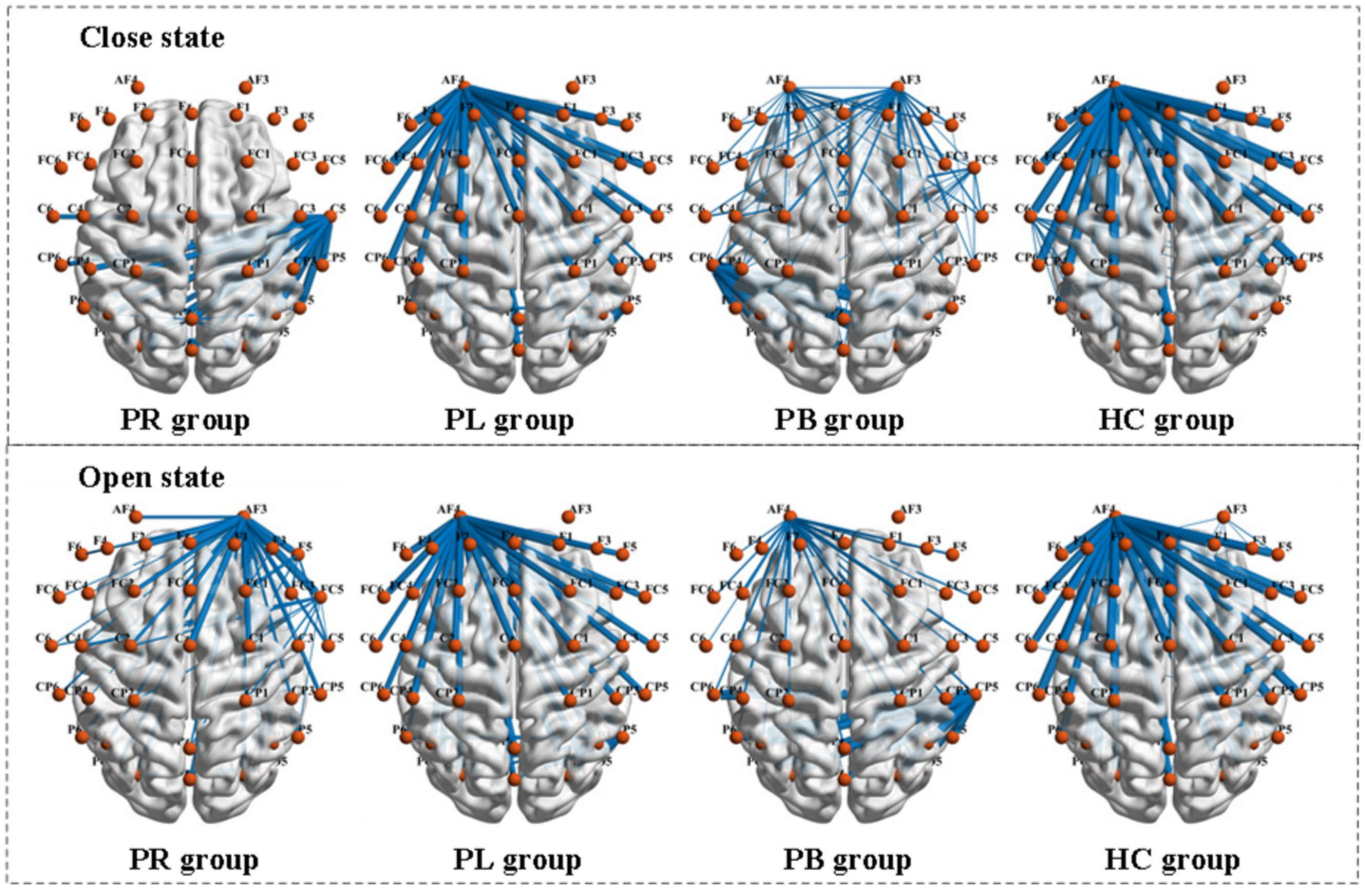
Comparison of PDC metrics between group mean channels in the PR, PL, PB, and HC groups in the closed-eye and open-eye states.

PLV shows whether there is a connection between the two channels, but which affects which cannot be obtained from the simultaneity analysis alone. So, we analyze the relationship between the two channels in terms of causality. Figure 9 shows the PDC values between the group average channels of the four groups in the closed-eye and open-eye state. A value of PDC close to 0 indicates that there is no connection between the two electrodes, while a value greater than 0.1 is considered a connection between the two channels. All channel combinations with PDC values greater than 0.156 are plotted in Figure 9. From the figure, it can be seen that in the closed-eye condition, there is a difference between the PB group and the other three groups, and there is also a difference and more significant between the PR group and the other three groups. However, there was no significant difference between the PL and HC groups. In the open-eye condition, there was no significant difference between the four groups. We further found that the PDC values between the AF4 channel and other channels were larger in both the PL and HC groups in the open-eye and closed-eye states. The PDC values between the C5 channel and other channels were larger in the PR group in the closed-eye state; and the PDC values between the AF3 channel and other channels were larger in the open-eye state. In the PB group, the PDC values between the CP6 channel and the other channels were larger in the closed-eye state; when the eyes were open, the PDC values between the CP5 and the other channels were larger. In other words, the calculation of PDC values may only be used to distinguish stroke patients with right-sided brain injury from healthy individuals, and may not be able to distinguish between other regions of brain injury.

### 4.3 Multi-modal features analysis

From the two parts A, B, it can be obtained that the features extracted from the EEG signals collected in the closed-eye state in all four groups of subjects have more significant differences than those in the open-eye state. In this paper, we propose to fuse the WPT features with FC features, and the form of the fused features is exactly the same as the previous FC features, represented as multi-modal (WPT + FC) features. The form of the fused multi-modal features is identical to the previous FC features, all of which are a square array measuring 39 × 39. The difference is that for each row of the square array of multi-modal features, the value of each element is the product of the value of the element at the same position of the FC feature and the value of the WPT feature of the electrode corresponding to that row, i.e., the value of the element in row *i* and column *j* of the square array of multi-modal features is the product of the value of the FC between electrodes *i* and *j* and the value of the WPT feature of the *i*-th electrode. For each row of the multi-modal features matrix, if the value of the element in that row is larger than the other rows, it means that the value of the WPT feature of the channel corresponding to that row is larger; if the value of some elements in that row is larger than the other elements in that row, it means that the channel corresponding to the column in which those elements are located has a larger FC index than the other channels. Thus, information on both FC features and WPT features can be obtained from the square matrix of multi-modal features.

**Figure 10:**
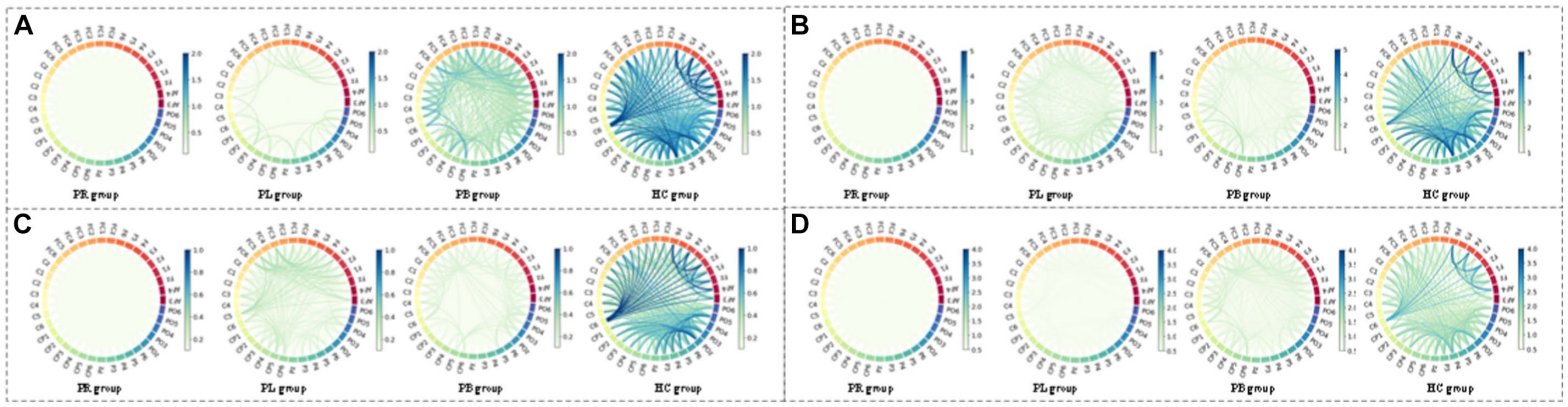
Multi-mode (WPT + FC) characteristic circle plots of wavelet energy ratio of α_1_, α_2_, β_1_ and β_2_ frequency bands fused with PLV in the closed-eye state. Where **(A)** α_1_ frequency band energy ratio + PLV; **(B)** α_2_ frequency band energy ratio + PLV; **(C)** β_1_ frequency band energy ratio + PLV; **(D)** β_2_ frequency band energy ratio + PLV.

**Figure 11:**
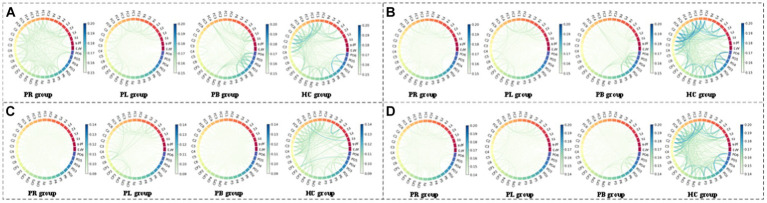
Multi-mode (WPT + FC) characteristic circle plots of WEE of α_1_, α_2_, β_1_ and β_2_ frequency bands fused with PLV in the closed-eye state. Where **(A)** α_1_ frequency band WEE + PLV **(B)** α_2_ frequency band WEE + PLV **(C)** β_1_ frequency band WEE + PLV **(D)** β_2_ frequency band WEE + PLV.

**Figure 12:**
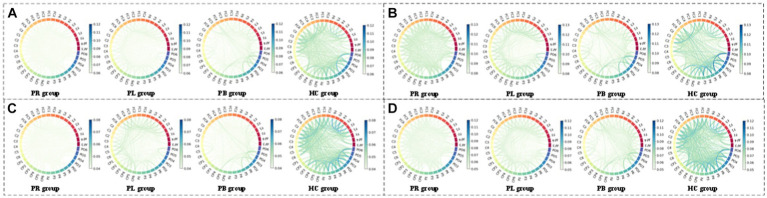
Multi-mode (WPT + FC) characteristic circle plots of WSE of α_1_, α_2_, β_1_ and β_2_ frequency bands fused with PLV in the closed-eye state. Where **(A)** α_1_ frequency band WSE + PLV **(B)** α_2_ frequency band WSE + PLV **(C)** β_1_ frequency band WSE + PLV **(D)** β_2_ frequency band WSE + PLV.

From subsection A, we obtained that in the closed-eye condition, there were significant differences between the PR, PL, PB and HC groups in the α_1_, α_2_, β_1_, and β_2_ frequency bands (*p* < 0.01); while in the δ and θ frequency bands, the differences between groups were smaller. From subsection B, we can get that the PLV index is more effective for distinguishing between PR, PL, PB and HC groups than the PDC index. Thus, in Figure 10, we show the circle plots of the four multi-modal features obtained by multiplying the wavelet energy ratio of the α_1_, α_2_, β_1_, and β_2_ frequency bands with PLV for subjects in the PR, PL, PB, and HC groups in the closed-eye state. For each feature, we set the feature values between channels displayed in the circle plot according to the values in the matrix. As can be seen from the figure, the value of the multi-modal features in the HC group are maximum in all four features, which is the same conclusion obtained in subsection A. The energy ratio in the higher frequency bands is significantly higher in healthy subjects than in stroke patients, which also shows that the features obtained after we performed feature fusion also retain information on the energy ratio in the frequency bands. In Figures 11, 12, we show the circle plots of the four multi-modal features obtained by multiplying the WEE and WSE of the α_1_, α_2_, β_1_, and β_2_ frequency bands with PLV for subjects in the four groups in the closed-eye state. As can be seen from the figure, stroke patients differ significantly from healthy subjects in the values of multi-modal features; the effect of differentiating regions of brain injury in patients we will further compare by categorical analysis. Meanwhile, from the diagram we can see more clearly which two poles have more significant related to each other.

### 4.4 Classification analysis and discriminant analysis

To investigate whether multi-modal features can be further used to differentiate regions of stroke injury, we designed two controlled trials with subjects in a closed-eye state. Based on 14 WPT features and 2 FC features, we were able to obtain 28 different sets of multimodal features. The results of the first set of controlled experiments are shown in Table 2, where the input was either these 28 sets of multi-modal features, or the sum of these 28 features. Table 2 shows which part of the data these 28 features were obtained from and the accuracy of distinguishing between the PR, PL, PB, HC groups. A comparison of the accuracy rates in Table 2 shows that the accuracy rates obtained for the multi-modal features in groups 13, 15, 17, 19, 21, 23, 25, and 27 are much higher than those obtained for the other groups, and even higher than the accuracy rates obtained for the multi-modal features in all groups. We refer to these eight groups of features collectively as multi-modal frequency features. This suggests that for the available data, the use of multi-modal frequency features in subjects’ resting-state data during closed-eye can be effective in detecting regions of stroke injury and distinguishing them from normal subjects.

**Table 2:** Results of accuracy classification analysis of multi-modal features.

Number	Multi-modal features		Accuracy (%)	Number	Multi-modal features		Accuracy (%)
	WPT	FC Feature			WPT Feature	FC Feature	
0	δ	PDC	56.49	14	$\alpha_{2}\mathrm{WEE}$	PDC	76.29
1	δ	PLV	46.84	15	$\alpha_{2}\mathrm{WEE}$	PLV	98.57
2	θ	PDC	67.56	16	$\beta_{1}\mathrm{WEE}$	PDC	78.75
3	θ	PLV	70.13	17	$\beta_{1}\mathrm{WEE}$	PLV	97.50
4	$\alpha_{1}$	PDC	75.53	18	$\beta_{2}\mathrm{WEE}$	PDC	77.56
5	$\alpha_{1}$	PLV	83.22	19	$\beta_{2}\mathrm{WEE}$	PLV	97.08
6	$\alpha_{2}$	PDC	80.21	20	$\alpha_{1}WSE$	PDC	80.53
7	$\alpha_{2}$	PLV	84.23	21	$\alpha_{1}WSE$	PLV	97.75
8	$\beta_{1}$	PDC	78.70	22	$\alpha_{2}WSE$	PDC	82.21
9	$\beta_{1}$	PLV	82.09	23	$\alpha_{2}WSE$	PLV	98.23
10	$\beta_{2}$	PDC	87.01	24	$\beta_{1}WSE$	PDC	84.70
11	$\beta_{2}$	PLV	90.73	25	$\beta_{1}WSE$	PLV	98.09
12	$\alpha_{1}\mathrm{WEE}$	PDC	77.13	26	$\beta_{2}WSE$	PDC	77.01
13	$\alpha_{1}\mathrm{WEE}$	PLV	97.75	27	$\beta_{2}WSE$	PLV	**99.75**
ALL	ALL	ALL	85.05				

To further investigate the difference in accuracy between feature fusion and individual features, we further designed a second set of control trials. In this group of control trials, the inputs are the individual frequencies of the multi-mode frequency features with high accuracy in the first set of control experiments, respectively. The results of the second set of control experiments are shown in Table 3. Compared to the classification results in Table 2, we obtain that the accuracy of the classification using a fusion of two features is higher than that of the classification using a single feature. This demonstrates that the optimal combination of features can improve the classification accuracy.

**Table 3:** Comparison between features.

Number	Feature	Accuracy (%)	Number	Feature	Accuracy (%)
0	PLV	85.97	5	$\alpha_{1}WSE$	81.25
1	$\alpha_{1}\mathrm{WEE}$	80.25	6	$\alpha_{2}WSE$	85.75
2	$\alpha_{2}\mathrm{WEE}$	85.50	7	$\beta_{1}WSE$	89.25
3	$\beta_{1}\mathrm{WEE}$	82.75	8	$\beta_{2}WSE$	90.00
4	$\beta_{2}\mathrm{WEE}$	86.50			

## 5 Discussion

We characterized EEG rhythms of right-sided brain-injured, left-sided brain-injured, and bilateral brain-injured stroke patients and healthy controls using time-frequency analysis and brain network analysis in the resting state with closed-eye and open-eye. Previous studies have generally concluded that altered EEG rhythms are present in the brains of stroke patients (Djamal et al., 2019, 2020; Wijaya et al., 2015). Our study showed that δ and θ bands were higher in the EEG signal of stroke patients, while α and β bands were reduced compared to healthy controls. Stroke-induced slowing and reduction in the complexity of the EEG signal. In addition, by analyzing the functional connectivity in the PR, PL, PB, HC groups, we provided new perspectives and insights into the phase differences between channels as well as information flow. We used a classifier to examine the ability of different features to discriminate between the closed-eye states, and to identify the best set of multi-model features as potential indicators to discriminate between regions of brain injury in stroke patients. We extracted 14 WPT features and 2 FC features to assess the variability, complexity and non-linear phase coupling information of frequency components in patients from different regions of brain injury. Finally, the maximum accuracy of the multi-modal frequency features in differentiating regions of brain injury was 99.75% in the closed-eye state.

Compared with healthy controls, the PR, PL, PB groups showed a significant increase in the energy ratio of δ and θ bands, and a significant decrease in both α (α_1_, α_2_) and β (β_1_, β_2_) bands, which is consistent with previous findings (Finnigan et al., 2016; Göksu, 2018; Djamal et al., 2019, 2020), where high-frequency bands decayed and slow-frequency bands became dominant. This suggests that the brains of stroke patients exhibit slower physiological behavior. These abnormalities may reflect pathophysiological changes: reduced energy occupancy at higher frequencies may be related to altered cortico-cortical connections; increased energy ratio at lower frequencies may be related to impaired cortical cholinergic pathways. Among the changes in the energy ratio of these six frequency bands, the most pronounced changes were found in the β_2_ band, followed by the β_1_ band. We also concluded that the energy ratio of each brain region in beta1 and beta2, differed significantly between the four groups. A previous study has extracted the β-band as a significant feature to classify stroke patients and healthy controls, obtaining 90% accuracy (Djamal et al., 2020). In addition, we calculated the mean whole-brain wavelet energy entropy and wavelet singular entropy for the 39 EEG channels in the PR, PL, PB, and HC groups in the open-eye and closed-eye states by analyzing the whole-brain WEE and the whole-brain WSE (Figure 5). We conclude that the mean values of wavelet entropy obtained in the open-eye state are greater than in the closed-eye state, indicating that the EEG signals collected by the subjects in the open-eye state are more complex. We obtained whole-brain-averaged WEE and WSE for the classification of regions of brain injury: with open-eye, the accuracy was 52.18% of WEE and 64.21% of WSE; with closed-eye, the accuracy was 53.29% of WEE and 69.87% of WSE. The formulas of WEE and WSE show that the magnitude of the entropy value is not only determined by the characteristics of the signal itself, but also related to the energy distribution of the signal in each frequency band. We further calculated the WEE and WSE of α_1_, α_2_, β_1_, and β_2_ frequency band. Table 3 gives the ability of WEE and WSE of these frequency bands to distinguish between regions of brain injury. The accuracy of WEE and WSE of α_1_, α_2_, β_1_ and β_2_ frequency band is higher compared to the accuracy of WEE and WSE averaged over the whole brain. A non-linear approach was applied to study brain activity in brain-injured patients and reduced complexity was found in brain-injured patients.

From a signal processing perspective, the EEG produces a non-linear and non-smooth representation of multivariate underlying neural circuit interactions. To effectively deal with non-stationarity, the EEG signals of 39 channels were projected into the time-frequency domain by wavelet packet transform and a set of WPT features were extracted; for the non-linear phase coupling information of the EEG signals, we used brain network analysis and extracted FC features to measure the degree of dependence and correlation between channels. The analysis of phase-locked values revealed that the strongest inter-channel connections were found in patients with right-sided brain injury stroke. Significant differences in phase-locked values were also observed between the four groups. The study of phase-locked values based on EEG signals can help one to understand the mechanisms of higher cognitive functions in the brain; whereas PDC values did not differ significantly between the four groups. This may be because there was less information flow between electrodes in the resting state for subjects to make significant differences. In order to confirm which of the WPT features or FC features can better distinguish between regions of brain injury, we extracted these features from the EEG signal for classification studies in later work.

A previous study stitched continuous wavelet transform features and bi-spectrum feature of EEG signals to achieve fusion of the two types of features to classify Alzheimer’s patients and healthy controls, and obtained higher accuracy than one type of feature alone (Zhang et al., 2012). However, this splicing method may increase the number of features and thus cause the problem of overfitting. In this paper, the multi-modal frequency features that we obtain do not increase the number of features. The values on the main diagonal of the multi-modal frequency features correspond to the values of the wavelet energy entropy and wavelet singular entropy of the corresponding channels, so that the multi-modal frequency features can characterize the time-frequency information. Since the multi-modal frequency features is a symmetric matrix, the size of the two symmetric values in the matrix depends not only on the complexity of the EEG signal in their respective channels, but also on the strength of the correlation between the two corresponding channels. This also suggests that this value in the multi-modal frequency features will only be used as an important feature if the correlated intersection between the two channels is strong and the complexity of the respective channel is also strong.

Finally, the fused multi-modal features from this paper were applied to classify the regions of brain injury in stroke patients. The results in Tables 2, 3 evaluate the classification performance of multi-modal features and single feature for the four groups of subjects, respectively. The results show that the classification performance of multi-modal frequency features in the closed-eye state is the best, with an accuracy of 99.75%. In previous studies, many studies have been conducted to classify stroke patients and healthy controls from the perspective of spectral features, complexity features and brain network features, but the accuracy obtained was low: 90% accuracy was obtained using β- band for classification (Djamal et al., 2020); 86.1% F_1_ score was finally obtained using 1D CNN to extract time-frequency features for classification (Giri et al., 2016); classification using power spectrum absolute and relative densities, spectral entropy and inter-channel coherence obtained an accuracy of 85% (Vivaldi et al., 2021). A comparative study found that the multi-modal frequency features proposed in this paper could not only improve the classification accuracy of stroke patients and healthy controls, but also classify regions of brain injury in stroke patients. In addition, for whether the subjects’ eyes were open or close, a previous study analyzed the functional activation of patients and normal controls in several brain regions by using resting-state functional MRI images and found that the difference in functional activation in these brain regions between patients and normal controls was more significant in the closed-eye condition (Hyvärinen and Oja, 2000).

Although the proposed method has good potential for application, the generalization performance needs to be further validated in the future due to the limited number of stroke patients. Therefore, future work will focus on researching domain-specific sample enhancement strategies to achieve quality augmentation of small sample datasets and further evaluate the generalization ability of the proposed method.

## 6 Conclusion

In this paper, we detected differences in cortical EEG signals in stroke patients with different brain damage regions by time-frequency analysis and brain network analysis. We extracted 14 WPT features and 2 FC features, then fused them for new feature, and classified patients with different regions of brain injury in resting state with closed-eye. Our results demonstrated that the energy ratio of δ and θ bands increased, while the energy ratio of α and β bands decreased in stroke patients compared to healthy controls, with the degree of both increase and decrease varying by region of brain injury. Additionally, we find that the complexity of the EEG signal was decreased in the α_1_, α_2_, β_1_ and β_2_ frequency bands of stroke patients, especially in the right-sided brain injury stroke patients. Furthermore, the PLV index showed high accuracy in distinguishing regions of brain injury, and all resulting 28 multi-modal frequency features (except δ band and θ band) could distinguish PR, PL, PB, and HC, of which having the highest accuracy of 99.75%. In summary, multi-modal frequency features can be used as a potential indicator to distinguish regions of brain injury in stroke patients.

## Data availability statement

The raw data supporting the conclusions of this article will be made available by the authors, without undue reservation.

## Ethics statement

The studies involving humans were approved by Ethics Committee of Dongyang People’s Hospital. The studies were conducted in accordance with the local legislation and institutional requirements. The participants provided their written informed consent to participate in this study.

## Author contributions

YJ: Data curation, Methodology, Visualization, Writing – original draft. JL: Methodology, Writing – original draft, Conceptualization, Data curation. ZF: Methodology, Writing – original draft, Visualization. XH: Methodology, Visualization, Data curation, Writing – original draft. TW: Conceptualization, Funding acquisition, Writing – review & editing, Visualization. SD: Conceptualization, Funding acquisition, Resources, Supervision, Writing – review & editing. XX: Conceptualization, Funding acquisition, Project administration, Resources, Supervision, Writing – review & editing. LL: Conceptualization, Resources, Supervision, Writing – review & editing.

## References

[ref1] Ang K. K. Chin Z. Y. Zhang H. Guan C. (2008). “Filter bank common spatial pattern (FBCSP) in brain-computer interface,” in 2008 IEEE international joint conference on neural networks (IEEE world congress on computational intelligence). IEEE.

[ref2] Cabin R. J. Mitchell R. J. (2000). To Bonferroni or not to Bonferroni: when and how are the questions. Bull. Ecol. Soc. Am. 81, 246–248.

[ref3] Djamal E. C. Gustiawan D. P. Djajasasmita D. (2019). Significant variables extraction of post-stroke EEG signal using wavelet and SOM kohonen. Telkomnika (Telecommunication Computing Electronics and Control) 17, 1149–1158. doi: 10.12928/telkomnika.v17i3.11776

[ref4] Djamal E. C. Ramadhan R. I. Mandasari M. I. Djajasasmita D. (2020). Identification of post-stroke EEG signal using wavelet and convolutional neural networks. Bull. Electr. Eng. Inform. 9, 1890–1898. doi: 10.11591/eei.v9i5.2005

[ref5] ElGhany S. A. Ibraheem M. R. Alruwaili M. Elmogy M. (2021). Diagnosis of various skin Cancer lesions based on fine-tuned ResNet50 deep network. Comp. Mater. Continua 68, 117–135. doi: 10.32604/CMC.2021.016102

[ref6] Fan Z. Xi X. Gao Y. Wang T. Fang F. Houston M.. (2023). Joint filter-band-combination and multi-view CNN for electroencephalogram decoding. IEEE Trans. Neural Syst. Rehabil. Eng. 31, 2101–2110. doi: 10.1109/TNSRE.2023.3269055, PMID: 37083516

[ref7] Finnigan S. Wong A. Read S. (2016). Defining abnormal slow EEG activity in acute ischaemic stroke: Delta/alpha ratio as an optimal QEEG index. Clin. Neurophysiol. 127, 1452–1459. doi: 10.1016/j.clinph.2015.07.014, PMID: 26251106

[ref8] Giri E. P. Fanany M. I. Arymurthy A. M. Wijaya S. K. (2016). “Ischemic stroke identification based on EEG and EOG using ID convolutional neural network and batch normalization,” in 2016 International conference on advanced computer science and information systems (ICACSIS). IEEE.

[ref9] Göksu H. (2018). BCI oriented EEG analysis using log energy entropy of wavelet packets. Biomed. Signal Process. Control 44, 101–109. doi: 10.1016/j.bspc.2018.04.002

[ref10] Huang W. Yan G. Chang W. Zhang Y. Yuan Y. (2023). EEG-based classification combining Bayesian convolutional neural networks with recurrence plot for motor movement/imagery. Pattern Recogn. 144:109838. doi: 10.1016/j.patcog.2023.109838

[ref11] Hyvärinen A. Oja E. (2000). Independent component analysis: algorithms and applications. Neural Netw. 13, 411–430. doi: 10.1016/S0893-6080(00)00026-5, PMID: 10946390

[ref12] Mahajan R. Morshed B. I. (2014). Unsupervised eye blink artifact denoising of EEG data with modified multiscale sample entropy, kurtosis, and wavelet-ICA. IEEE J. Biomed. Health Inform. 19, 158–165. doi: 10.1109/JBHI.2014.2333010, PMID: 24968340

[ref13] Pereda E. Quiroga R. Q. Bhattacharya J. (2005). Nonlinear multivariate analysis of neurophysiological signals. Prog. Neurobiol. 77, 1–37. doi: 10.1016/j.pneurobio.2005.10.003, PMID: 16289760

[ref14] Powers D. M. (2020). Evaluation: from precision, recall and F-measure to ROC, informedness, markedness and correlation. arXiv preprint arXiv:2010.16061.

[ref15] Röschke J. Aldenhoff J. (1992). A nonlinear approach to brain function: deterministic chaos and sleep EEG. Sleep 15, 95–101. doi: 10.1093/sleep/15.2.95, PMID: 1579794

[ref16] Sameshima K. Baccalá L. A. (1999). Using partial directed coherence to describe neuronal ensemble interactions. J. Neurosci. Methods 94, 93–103. doi: 10.1016/S0165-0270(99)00128-4, PMID: 10638818

[ref17] Savelov A. Shtark M. Mel’nikov M. Kozlova L. Bezmaternykh D. Verevkin E.. (2019). Prospects of synchronous fMRI-EEG recording as the basis for neurofeedback (exemplified on patient with stroke sequelae). Bull. Exp. Biol. Med. 166, 390–393. doi: 10.1007/s10517-019-04357-8, PMID: 30627899

[ref18] Schneider A. L. Jordan K. G. (2005). Regional attenuation without delta (RAWOD): a distinctive EEG pattern that can aid in the diagnosis and management of severe acute ischemic stroke. Am. J. Electroneurodiagnostic Technol. 45, 102–117. doi: 10.1080/1086508X.2005.11079517, PMID: 15989073

[ref19] Tsao C. W. Aday A. W. Almarzooq Z. I. Alonso A. Beaton A. Z. Bittencourt M. S.. (2022). Heart disease and stroke statistics—2022 update: a report from the American Heart Association. Circulation 145, e153–e639. doi: 10.1161/CIR.0000000000001052, PMID: 35078371

[ref20] Vivaldi N. Caiola M. Solarana K. Ye M. (2021). Evaluating performance of eeg data-driven machine learning for traumatic brain injury classification. IEEE Trans. Biomed. Eng. 68, 3205–3216. doi: 10.1109/TBME.2021.3062502, PMID: 33635785 PMC9513823

[ref21] Wijaya S. K. Badri C. Misbach J. Soemardi T. P. Sutanno V. (2015). “Electroencephalography (EEG) for detecting acute ischemic stroke,” in 2015 4th International conference on instrumentation, communications, information technology, and biomedical engineering (ICICI-BME). IEEE.

[ref22] Zhang H. Qu G. Ren T. (2012). Research on the detecting methods of singularity in deformation signal based on two kinds of wavelet entropy. J. Coal Sci. Eng. (China) 18, 213–217. doi: 10.1007/s12404-012-0219-4

